# Scalable and responsive event processing in the cloud

**DOI:** 10.1098/rsta.2012.0095

**Published:** 2013-01-28

**Authors:** Visalakshmi Suresh, Paul Ezhilchelvan, Paul Watson

**Affiliations:** School of Computing Science, Newcastle University, Newcastle upon Tyne NE1 7RU, UK

**Keywords:** event processing, queueing theory, analytical estimation, algorithms, experimental validation

## Abstract

Event processing involves continuous evaluation of queries over streams of events. Response-time optimization is traditionally done over a fixed set of nodes and/or by using metrics measured at query-operator levels. Cloud computing makes it easy to acquire and release computing nodes as required. Leveraging this flexibility, we propose a novel, queueing-theory-based approach for meeting specified response-time targets against fluctuating event arrival rates by drawing only the necessary amount of computing resources from a cloud platform. In the proposed approach, the entire processing engine of a distinct query is modelled as an atomic unit for predicting response times. Several such units hosted on a single node are modelled as a multiple class M/G/1 system. These aspects eliminate intrusive, low-level performance measurements at run-time, and also offer portability and scalability. Using model-based predictions, cloud resources are efficiently used to meet response-time targets. The efficacy of the approach is demonstrated through cloud-based experiments.

## 1. Introduction

Event processing is characterized by the continuous processing of streamed data tuples or *events* in order to evaluate, in a timely manner, the queries deployed by decision support systems. Event sources can, for example, be pervasive sensors. The rates at which these sources generate events can vary widely and often unpredictably, driven purely by the external processes they monitor. Similarly, the number of queries that need to be evaluated can also vary over time. Thus, an event-processing system with real-time performance requirements must meet targeted response times despite being subjected to these two types of varying loads.

A query evaluation can be modelled as a directed acyclic graph (DAG) wherein vertices are operators and edges are event streams that are either directly from sensors or partially processed streams from the preceding operators. Meeting response time targets despite varying arrival rates is a classical problem of load optimization [1–4]. In the context of event processing, the granularity of load optimization has been DAG vertices or a sub-graph of DAG.

Early systems, such as Aurora [5], identify operators common to multiple queries for efficient resource provisioning in a single server context. Later, distributed solutions [6,7] handled the optimization problem as a load-balancing issue over a fixed set of nodes: moving query operators to nodes where their resource requirements are best met. Such solutions require probes to measure operator execution rates, queue lengths, etc., making implementation hard and possibly not portable across heterogeneous machines; further, they may have to sacrifice targets for some queries to meet others' targets [7].

Recent work [4] pursues a parallel-distributed approach at the operator level by dividing DAG into sub-graphs. This intra-operator parallelism is similar to parallelization commonly applied in databases [1,3]. Although the available servers are not fixed in [4], the spare ones must be kept ‘warm’ by running a bespoke software.

In this paper, we take a coarse-grained approach to load optimization: the granularity is the state machine or the *event processing network* (EPN) that implements the entire DAG of a given query. Being coarse-grained has two advantages: variations in the number of queries to be processed can be easily dealt with, provided additional hosts are available; secondly, spare hosts need not be kept warm as low-level parallelization is not sought. These advantages make our approach best suited to using cloud platforms.

Being coarse-grained also poses new challenges that are addressed by modelling an EPN from the queueing theory perspective and by predicting response times as a function of event arrival rates. The rationale behind our modelling can be briefly explained as below (with details in §5).

Incoming streams of an EPN go through a sequence of query operators before triggering an output event. The output latency or the *response time* therefore consists of three major components:

(i) the wait time before encountering the first query operator in the sequence,
(ii) the wait time between query operators, and
(iii) the sum of query operator execution times.

When the arrival rate of tuples increases, wait time (i) is seriously affected, the inter-operator delay (ii) is less affected and the operator execution times (iii) are least affected. When more than one EPN are hosted by a single host, (ii) is impacted owing to competition for CPU usage. On the basis of these observations, we model an EPN as a single queue ‘server’ system wherein the server is the composite operator consisting of all operators within that EPN. The waiting time in the queue models (i) and the *processing time* by the ‘server’ models the sum of (ii) and (iii). We use queueing theory to predict (i) and off-line calibration to establish (ii) and (iii).

The paper is organized as follows. Section 2 describes the event processing system that must meet response time targets when arrival rates can vary unpredictably. Section 3 presents the overall architecture, and §4 highlights the role of the configuration scheduler (CS) in mapping EPNs to hosts based on the performance targets of the former and the processing capacities of the latter. Sections 5 and 6 present the queueing theory-based models and the algorithm for mapping EPNs to hosts, respectively. Experimental results are presented in §7 and §8 concludes the paper.

## 2. System description

The system processes multiple event streams, each emanating from a unique source (e.g. a sensor device). These streams are denoted as *s*_1_,*s*_2_,*s*_3_,…,*s*_*σ*_ and the set of all event streams entering the system is defined as *Σ*=*s*_1_,*s*_2_,…,*s*_*σ*_. The system evaluates *q* queries, *Q*_1_,*Q*_2_,…,*Q*_*q*_. The state machine that implements the DAG for *Q*_*i*_ is called the EPN_*i*_. Evaluating *Q*_*i*_ involves processing one or more event streams and the set of all streams input to EPN_*i*_ is denoted as *S*_*i*_. Note that *S*_*i*_ of EPN_*i*_ and *S*_*j*_ of EPN_*j*_ may overlap. Also, an input stream to EPN_*i*_ can be an output stream from another EPN_*j*_; if so, *S*_*i*_∉*Σ*. If all inputs to EPN_*i*_ are output streams from other EPNs, then *S*_*i*_∩*Σ*={}.

An EPN is also associated with a performance target *T*. It is said to be distinct if any one of its three attributes is unique: DAG, *S* or *T*. We consider each EPN to be distinct. The system itself is made up of *n* hosts or virtual machines, denoted here generically as *nodes*, drawn from a cloud computing platform. The number of nodes used, *n*, is increased (or decreased) when the load increases (or decreases) to an extent that the current configuration over these *n* nodes is deemed inadequate (or more than strictly necessary, respectively), to meet the performance targets.

**Figure 1.**
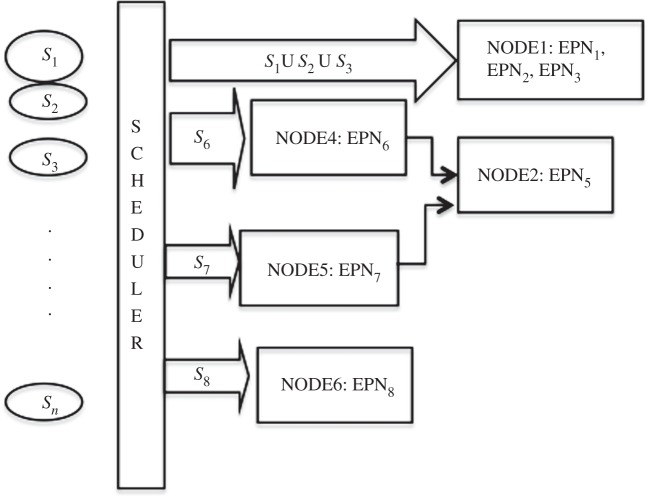
The event processing architecture.

A *configuration* is a mapping from the set of EPNs onto the set of hosts. Figure 1 shows a configuration where EPN_1_, EPN_2_ and EPN_3_ are mapped to (i.e. hosted by) node 1, and the rest of the EPNs are mapped to a distinct node. The system has a CS, which decides the configuration appropriate to the load conditions and performance targets associated with the EPNs. For brevity, we assume that the CS is centralized, hosted on a single node. Its role and workings are discussed in §§3 and 4, respectively.

## 3. The architecture

The front end of our system has a scheduler that forwards the incoming streams to processing nodes according to the policy decided by the CS. Stream forwarding could be done through MOM (message-oriented middleware) or through an asynchronous socket service. When CS announces a new EPN–host mapping, i.e. a new configuration, each processing node subscribes to relevant input streams and transmits its relevant output streams to nodes of EPN which need them as inputs.

Central to our architecture is the CS whose design is described in §4. In a nutshell, each EPN takes macro-level measurements of its own performance and reports periodically to CS, which constructs a global view and attempts to re-map EPNs to host nodes, if response times of some EPNs are either larger or far smaller than their target levels; in the former case, new nodes may have to be brought in; in the latter case, some of the nodes may be freed from use. Note that re-mapping of EPNs to hosts extracts a cost that is not measured here; rather, the focus will be on our design of CS and on evaluating the effectiveness with which appropriate configurations are chosen so that response-time targets are met.

## 4. Configuration scheduler

Each node monitors, for every EPN_*i*_ that it hosts, the response times and the sum of arrival rates of streams input to that EPN_*i*_; the average response time and the largest total arrival rate observed for EPN_*i*_ over the reporting interval are recorded as RT_*i*_ and AR_*i*_ respectively, and are reported to CS at the end of each interval. For example, *Node*_1_ in figure 1, which hosts EPN_1_, EPN_2_ and EPN_3_, will report to CS {RT_1_,AR_1_}, {RT_2_,AR_2_} and {RT_3_,AR_3_}.

Let *T*_*i*_ denote the average response-time target specified for any given EPN_*i*_. We define
4.1
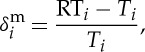
where 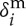 is the *relative deviation* of RT_*i*_ and m indicates that 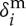 is measurable.

We define lower and upper bounds for 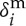: *low water-mark* and *high water-mark*, denoted as LW and HW, respectively. If 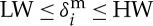, then EPN_*i*_ is deemed to meet its performance target within the scope of chosen LW and HW.

If all *q* EPNs in the system are deemed to meet their respective targets, then the current configuration is considered to be working well and CS does nothing; otherwise, CS decides on a new configuration by dividing *q* EPNs into *ζ* disjoint sets, *Z*_1_,*Z*_2_,…,*Z*_*ζ*_, and by ensuring that the following two constraints are met when all EPNs of every given *Z*_*x*_, 1≤*x*≤*ζ*, are hosted within a unique node:

1. *ζ* is the smallest possible, and
2. 
  (a) LW≤*δ*^m^≤HW holds for each EPN in the system, and
  (b) The total load exerted by the EPNs of every *Z*_*x*_ does not exceed a load threshold, *ρ*_th_≤1.

These two constraints make the new configuration decided by the CS an optimal one. Meeting the first constraint becomes one of *optimal assignment* problem, provided that 2a and 2b can be analytically evaluated. To evaluate 2a and 2b for a given configuration choice, we derive formulae to *estimate* the average response times and the CPU load. If estimations are accurate, then the evaluations of 2a and 2b will be accurate. Derivations of estimation formulae are discussed in §5, and their accuracy is experimentally assessed in §7.

The optimal assignment problem can be NP-complete; hence, we solve it by using the well-known heuristic algorithm known as the *bin-packing* algorithm. The algorithm packs the EPNs into the smallest number of nodes (bins), subject to conditions 2a and 2b. This heuristic algorithm is presented in §6.

## 5. Analytical estimation of event processing network response times and CPU usage

Analytical estimations make a simplifying assumption that a node can host any single EPN on its own *and* satisfy both 2a and 2b. If this assumption does not hold, then intra-EPN parallelism, similar to intra-operator parallelism used in [4], would be necessary. (See also remarks made at the end of this section.)

Let us suppose that a node hosts the EPNs of some *Z*_*x*_={EPN_1_,EPN_2_,…,EPN_*k*_}. Estimations for this scenario are done in two parts: (i) modelling a single EPN_*i*_∈*Z*_*x*_ as a single-queue, single-server system and then (ii) modelling all EPNs of *Z*_*x*_ as a M/G/1 multi-class queueing system.

### (a) Modelling and calibrating a single event processing network

**Figure 2.**
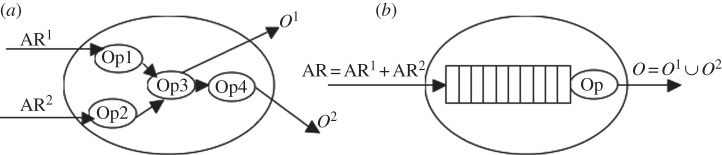
(*a*) Internals of an EPN. (*b*) Single-server, single-queue model of the EPN.

Figure 2*a* presents the DAG structure of a simple EPN chosen as an example. There are two input streams with arrival rates AR^1^ and AR^2^. After they are acted on by distinct operators (Op1 and Op2), they are joined at Op3, which produces an output stream *O*^1^ to the environment and an input stream to Op4 which then generates *O*^2^. Note that each Op would have its own buffer to store the incoming events, which are not shown in figure 2*a*.

Irrespective of its DAG structure, the EPN of figure 2*a* is modelled as a single server system that receives a single input stream with arrival rate AR=AR^1^+AR^2^, and generates a single output stream O = *O*^1^∪*O*^2^; the incoming event tuples are queued, and the tuple at the head of the queue is processed by a super operator OP composed of Op1, Op2, Op3, Op4 (figure 2*b*). The tuples that get past the head of the queue, in our model, are ‘processed’ by OP as per the logic of the EPN of figure 2*a* and generate *O*=*O*^1^∪*O*^2^.

We apply the above modelling approach to estimate the average response time of, and the CPU load exerted by, any single EPN. First, we define or recall a few metrics, some of which are measured dynamically and the rest established through off-line calibration. Let EPN_*i*_ be any EPN in the system.

*Event arrivals* denote the arrivals of events of streams in *S*_*i*_ and are taken to be Poisson at the rate of AR_*i*_ that is supplied to CS at the end of each reporting interval.

*Processing time* denotes the total processing time a tuple or a window of tuples needs to undergo to produce an output *O*^*j*^∈*O* after a tuple in the window has just gone past the head of the queue as in figure 2*b*. Note that it does not include the time that a tuple spends between its arrival and reaching the head of the queue, i.e. the *queueing delays*. Moreover, the processing time depends on the nature of DAG_*i*_ that EPN_*i*_ implements and also on the particular path that a tuple takes within DAG_*i*_ for it to be processed and an appropriate output to be generated.

Given the non-deterministic nature of tuple-flows, the processing time is modelled as a random variable of some unknown distribution. When EPN_*i*_ generates several outputs, the one that takes the maximum processing time will be of our interest. For this output, we define *b*_*i*_ as the *average processing time* and *M*_2,*i*_ as the *second moment* of the processing times.

*Processing load* imposed by EPN_*i*_ on the host node is *ρ*_*i*_. Specifically, *ρ*_*i*_ is the fraction of the time EPN_*i*_ used the node's CPU. Thus, *ρ*_*i*_=AR_*i*_×*b*_*i*_ and this relation is used to establish *M*_2,*i*_ and *b*_*i*_ through *calibration* as described below.

EPN_*i*_ is hosted on its own on a node and subject to ‘small’ arrival rates AR_*i*_ (to eliminate or at least minimize queueing delays); the CPU usage for each rate is observed and the resulting processing times are also computed from the observations. From these times, the most processing intensive output is identified, and *M*_2,*i*_ and *b*_*i*_ are established. EPNs typically process input events in groups or windows of, say, *w* tuples; if so, the arrival rate during calibration should not exceed *w* events per second.

### (b) Modelling multiple event processing networks in a single host

Recall that a single node hosts *k* EPNs of *Z*_*x*_. We abstract this collection of EPNs in the same way as we abstracted the collection of operators of a single EPN; more precisely, the *k* EPNs of *Z*_*x*_ are replaced by a *composite* EPN and the input events of various EPNs are placed in a single FIFO queue; when an event or a window of appropriate events gets past the head of the queue, the (virtual) processor of the node processes it by executing the appropriate EPN_*i*_. The model thus becomes a M/G/1 multiple class queueing system [8].

Using the well-known M/G/1 results, we next derive expressions for metrics of interest, when a node hosts *k* EPNs of *Z*_*x*_={EPN_1_,EPN_2_,…,EPN_*k*_}. Note that the total arrival rate (AR) at the node is the sum of the arrival rates of the *k* hosted EPNs (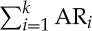); similarly, the total load (*ρ*) on the node is the sum of the load exerted by every EPN_*i*_∈*Z*_*x*_. Thus,
5.1
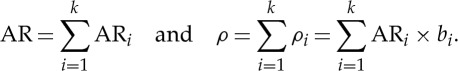


We denote the average response time estimated for any EPN_*i*_∈*Z*_*x*_ as *W*_*i*_:
5.2
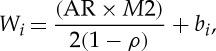
where 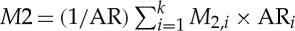. Similar to 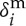 defined by expression (4.1), we let 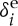 denote the *relative deviation* of *W*_*i*_, with e indicating that 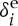 is based on estimation:
5.3
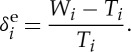


If *W*_*i*_ estimates RT_*i*_ reasonably accurately, 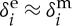, and 2a and 2b of §4 can be evaluated; more precisely, given that the EPNs are divided into *ζ* disjoint sets, *Z*_1_,*Z*_2_,…,*Z*_*ζ*_, using a unique node to host each *Z*_*x*_, 1≤*x*≤*ζ*, leads to a viable configuration, if
5.4
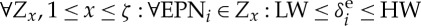
and
5.5
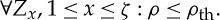

**Remarks**

Condition (5.4) states that every EPN in every *Z*_*x*_ must have its estimated deviation within the water-marks, and condition (5.5) requires that the total load imposed on every *Z*_*x*_ must not exceed *ρ*_th_. They verify 2a and 2b of §4, respectively, provided that each node being used for hosting a set of EPNs is (i) a single CPU or a single core machine and (ii) has a CPU that is as powerful as the CPU of the one used for the calibration of EPNs.

If the CPUs of the host nodes are more powerful, the actual response times would be smaller than the estimates and hence the evaluation of the conditions (5.4) and (5.5) would be pessimistic and would result in more nodes being used than strictly necessary. On the other hand, if the calibration has been done using nodes with more powerful CPUs, response-time targets may be missed frequently.

In intra-EPN parallelism, a given EPN_*i*_ is hosted on multiple nodes, and each input stream in *S*_*i*_ is temporally split and each split is input to one of the hosting nodes. For example, let EPN_*i*_ be hosted by two nodes as 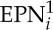 and 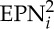; an input stream *s*_*i*_∈*S*_*i*_ can be split as: first 100 tuples (1–100) as 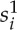, the next 100 tuples (101–200) as 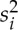, the next 100 tuples (201–300) as 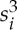, and so on. Odd splits, (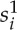, 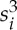, …, 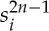, …), are sent to 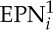 (in order) and even splits to 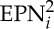, thus halving the arrival at each node. The results from 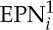 and 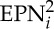 would have to be ‘reduced’ for the final result.

## 6. Selecting a new configuration

Each EPN_*i*_ in the system is represented within CS by five parameters: the measured, average response time RT_*i*_ (as periodically reported to CS), the target response time *T*_*i*_ (given), the measured, total arrival rate AR_*i*_ (reported to CS), the processing time *b*_*i*_ (calibrated) and an estimated response time *W*_*i*_ (using equation (5.2)).

When CS observes that one or more EPNs have their *δ*^m^ exceeding HW or falling below LW, it would seek a new appropriate configuration. This selection process itself does not require halting of any EPNs and can proceed in parallel; however, if it is decided that the new configuration be implemented, then some EPNs will inevitably have to be moved to different nodes; also, some event streams will have to be re-directed and some others may have to be duplicated and streamed to multiple nodes.

The algorithm used for determining the next, appropriate configuration is a heuristic one and works in three parts. In *part 1*, it builds three types of *ordered* lists that provide a structured global view on the performance status of EPNs and the total load on each host node. For every node *N*_*j*_, the EPN list EL_*j*_ contains all EPNs hosted by *N*_*j*_. The *donor list* DL consists of nodes that must give up some of the EPNs that they currently host, and the *acceptor list* AL has nodes that can possibly host additional EPNs.

*Step* 1.1. For every node *N*_*j*_, 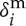 is computed for each EPN_*i*_ hosted by *N*_*j*_, and the EPN list EL_*j*_ is ordered in the non-increasing order of 
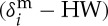
.

*Step* 1.2. If *N*_*j*_ is hosting no EPN_*i*_ with 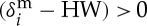, it is marked as an *acceptor* node and is entered in the acceptor list AL; otherwise, it is marked as a *donor* node and is entered in the donor list DL.

*Step* 1.3. DL is ordered in the non-increasing order of the total load (*ρ*) imposed on the nodes, while AL is ordered in the other way round: in the non-decreasing order of the total load on the nodes.

*Discussions*. At the end of part 1, DL will be empty if an execution of the algorithm has started because some EPNs had *δ*^m^<LW; similarly, AL will be empty if each node had some EPN with *δ*^m^>LW when the algorithm started. Both DL and AL cannot, however, be empty when part 1 completes.

The objective of *part 2* is to make a non-empty DL empty. Let *D* and *A* denote the first node in DL and AL, respectively. *D* is the most heavily loaded donor node, hosting at least one EPN whose *δ*^m^>HW; *A* is the most lightly loaded acceptor and hence it becomes the first candidate to be tried for the possibility of hosting an under-performing EPN in *D*. This forms the basis for part 2, which is skipped if DL is empty to start with.

*Step* 2.1. Let EPN_*D*_ be the EPN with the largest (*δ*^m^−HW) in EL_*D*_. Let EL_*A*_=EL_*A*_∪{EPN_*D*_}. Compute *Z*_*A*_={EPN_*i*_:EPN_*i*_∈EL_*A*_} and check if conditions (5.4) and (5.5) hold for *Z*_*A*_. If they hold, then execute step 2.2. If they do not, reset EL_*A*_=EL_*A*_−EPN_*D*_; if there is a node that is ordered immediately after *A* in AL, then set *A* to be that node and execute step 2.3; otherwise, execute step 2.4.

*Step* 2.2. Set EL_*D*_=EL_*D*_−EPN_*D*_ to indicate that EPN_*D*_ is to be moved from node *D* to node *A* in the new configuration. Note that the contents of EL_*D*_ and EL_*A*_ are changed and therefore *δ*^m^ computed (in step 1.1) for the EPNs in these two lists are no longer valid. So, execute sub-step *re-compute*(*δ*) for nodes *D* and *A*. Re-assess the donor status of node *D*; if the status of *D* is changed, update the lists DL and AL. Order the nodes of AL. If DL is not empty, order the nodes of DL, set *D* and *A* as the first node in DL and AL respectively, and execute step 2.1. If DL is empty, execute part 3.

*Sub-step* *re-compute*(*δ*). If *EL*_*j*_ of node *N*_*j*_ does not reflect the EPNs that *N*_*j*_ is hosting in the current configuration, then set *Z*_*x*_={EPN_*i*_:EPN_*i*_∈EL_*j*_}; for every EPN_*i*_∈*Z*_*x*_: compute 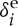 using the expression (5.3) and 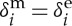; order the entries of EL_*j*_ in the non-increasing order of 
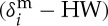
; return to the calling step.

*Step* 2.3. With the new *A*, execute step 2.1.

*Step* 2.4. Hire a new node from a cloud platform and call it *A*; initialize EL_*A*_ to {}; enter *A* as the first node in AL; execute step 2.1.

*Discussions*. Step 2.1 attempts to replace the mapping of EPN_*D*_ to *D* by trying to map EPN_*D*_ to *A*. If re-mapping is feasible, step 2.2 maps EPN_*D*_ to *A*; otherwise the next node in AL is tried in step 2.3. If AL has no suitable *A* at all for re-mapping EPN_*D*_, step 2.4 hires a new *A*. These steps are repeated until an EPN_*D*_ exists, i.e. until DL is not empty.

*Part* 3 attempts to reduce the size of AL, when the size is more than one. Recall that the first node in the ordered AL is the least-loaded and hence it is the first candidate to be tried for the possibility of being freed from use. It is called the *pseudo-donor* node and denoted as *D* in part 3. A new acceptor list called *deutero acceptor list*, DAL for short, is created as a copy of AL but without the first node in AL.

Let *D* and *A* be the first node in AL and DAL, respectively. (Note: *D* is not in DAL.) EPNs of *D* are tried, one by one, to be moved to some node in DAL. If all EPNs of *D* cannot be moved out, part 3 stops after restoring ELs of nodes in AL; otherwise, the new AL becomes DAL and the new DAL is the new AL without the first node. If the new DAL is not empty, part 3 is repeated; else, the algorithm stops.

*Step* 3.0. Discard earlier checkpoint, if any; checkpoint EL_*j*_ of every *N*_*j*_∈AL.

*Step* 3.1. Let EPN_*D*_ be the EPN with the largest (*δ*^m^−HW) in EL_*D*_. EL_*A*_=EL_*A*_∪{EPN_*D*_}. Compute *Z*_*A*_={EPN_*i*_:EPN_*i*_∈EL_*A*_} and check if conditions (5.4) and (5.5) hold for *Z*_*A*_. If they hold, then execute step 3.2. If they do not hold and if *A* is the last node in AL, restore EL_*j*_ of each *N*_*j*_∈AL and terminate the algorithm. If conditions (5.4) and (5.5) do not hold for *Z*_*A*_ and if *A* is not the last node in AL, reset EL_*A*_=EL_*A*_−EPN_*D*_, set new *A* to be the node next to the current *A* in DAL and re-execute step 3.1.

*Step* 3.2. Set EL_*D*_=EL_*D*_−EPN_*D*_. Execute sub-step *re-compute*(*δ*) for *A*. Order the nodes of DAL. If EL_*D*_ is not empty, then set *A* as the first node in DAL and execute step 3.1. If EL_*D*_ is empty, then AL=DAL and set new DAL. If DAL is not empty, set new *A* and new *D* and execute step 3.0. If DAL is empty, terminate the algorithm.

## 7. Validation

The objectives here are twofold: assess the accuracy of estimation formulae of §5 and the appropriateness of new configurations selected. EPNs used are *activity recognition engines* of the Newcastle Ambient Kitchen project [9] that aims to aid dementia patients in the midst of their kitchen activities. The purpose built kitchen is equipped with sensor-embedded kitchen utensils, such as spoons, forks, eating knives, chopping knives and so on.

EPNs process the signals from the sensors and recognize the activities that the source utensils are engaged in. Note that when activities in the kitchen increase, the arrival rates increase and *vice versa*. Recognitions from EPNs are passed to an intelligent system that identifies any unduly long pauses in the activity sequences and instructs the object holders accordingly. The intelligent system is not a part of our experiments.

In the terminology of §2, each of the input streams, *s*_1_,*s*_2_,*s*_3_,…,*s*_*σ*_ (see §2), emanates from a unique object in the ambient kitchen and *σ* can be around 600. In our experiments, however, only five EPNs were used as a proof of concept demonstration. Each EPN takes input from a unique sensor and is deployed as a small EC2 instance with 1.7 GB memory, 1EC2 compute unit, 160 GB instance storage, 32 bit platform with Ubuntu operating system.

An EPN processes events in the order of their generation and in units of 64. (This figure of 64 was deemed optimal as per the experiments in [5].) Each unit is called a window and consists of 32 new events appended with the last 32 events of the previous window. Thus, a sensor event is processed twice in two successive windows.

Each EPN has three operators: building windows of 64 events (Op1), mapping each window into an attribute tuplet (Op2) and comparing attribute tuplets with a template to recognize the activity of the event source (Op3). Event arrival rate peaks when the source is under maximum use and drops to zero when at rest. Thus, EPNs face a fluctuating arrival rate (assumed to be Poisson with rate AR_*i*_ in §4). In the experiments, traces of event streams recorded in real-life situations were sent at the rates of our choice.

Given the nature of event processing, we assumed that the processing times are exponentially distributed with mean *b*_*i*_. So, the second moment *M*_2,*i*_ simplifies to 2(*b*_*i*_)^2^, leaving only *b*_*i*_ to be calibrated, which was done as follows. For a range of small AR_*i*_<32 events per second, each EPN was run on a single node, and the load *ρ*_*i*_ imposed in each case was observed. The average of the values computed using *ρ*_*i*_/AR_*i*_ is taken as *b*_*i*_.

**Figure 3.**
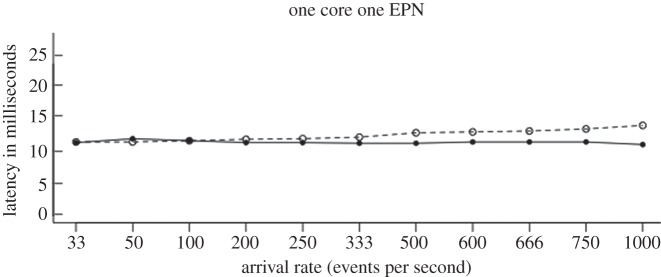
Response times measured (open circles) and estimated (filled circles).

Figure 3 plots the response times, measured (RT) and estimated (*W*), for various arrival rates common in the application context. *W*≈RT holds for arrival rates up to 250 and thereafter (*RT*−*W*) increases gradually. This is not surprising as our single-queue abstraction ignores inter-operator buffering delays. RT getting larger than *W* for higher arrival rates means that RT is likely to exceed *T* at these arrival rates. This can be catered for by appropriately adjusting *W*, a topic to be investigated in future.

### (a) Configuration selection

The efficacy of the algorithm for selecting new configurations is demonstrated by varying arrival rates of five EPNs at hourly intervals and presenting the configurations selected. The demonstration shows the following three aspects, while maintaining the capability to meet target response times even when a single node hosts multiple EPNs:

— shifting some EPNs among existing nodes;
— hiring more nodes when necessary; and
— releasing nodes when arrival rates decrease.

HW, LW and *ρ*_th_ are fixed to be 20%, −20% and 0.8, respectively. Reporting interval was fixed as 10 s: nodes reported RT and AR of every hosted EPN once every 10 s. Changes to arrival rates were administered as ‘triggers’ at hourly intervals.

CS was programmed to produce a new schedule only when triggers were applied; i.e. CS would not act whenever it observed *δ*^m^ for some EPN not satisfying LW≤*δ*^m^≤HW. The objective is to study the EPN performance when configuration is changed only in response to changes in AR; if that is sufficient, then proactive reporting would not be necessary and the reporting overhead can be reduced.

Before the first hour, *H*=1, AR of all five EPNs is zero, i.e. the system of EPNs was idle; AR_1_ was set to 500 (events per second) at the start of the first hour, i.e. at *H*=1.

The first two columns of table 1 present the triggers applied at various *H* values. Column 2 indicates the changes applied over what existed earlier; this means that when a trigger, say, at *H*=4 does not involve EPN_1_, this means that AR_1_ was not changed at *H*=4 and hence AR_1_ retains the value it had just before *H*=4, which is 1000.

**Table 1.** Configurations in response to triggers.

hour (*H*)	trigger nature	node 1	node 2	node 3	node 4	node 5
1	AR_1_ at EPN_1_=500	EPN_1_				
2	AR_2_ at EPN_2_=500	EPN_1_				
		EPN_2_				
3	AR_1_ at EPN_1_=1000	EPN_1_	EPN_3_			
	AR_2_ at EPN_2_=1000	EPN_2_	EPN_4_			
	AR_3_ at EPN_3_=1000					
	AR_4_ at EPN_4_=500					
4	AR_4_ at EPN_4_=1000	EPN_1_	EPN_3_	EPN_5_		
	AR_5_ at EPN_5_=1000	EPN_2_	EPN_4_			
5	AR_2_ at EPN_2_=2000	EPN_1_	EPN_3_	EPN_5_	EPN_2_	EPN_4_
6	AR_5_ at EPN_5_=0	EPN_1_	EPN_3_			
	AR_4_ at EPN_4_=500	EPN_2_	EPN_4_			
	AR_2_ at EPN_2_=1000					
7	AR_1_ at EPN_1_=500	EPN_1_	EPN_3_			
	AR_2_ at EPN_2_=500	EPN_2_	EPN_4_			
	AR_3_ at EPN_3_=500					
8	AR_1_ at EPN_2_=0	EPN_1_				
	AR_4_ at EPN_4_=0					
	AR_3_ at EPN_3_=0					

It can be seen from table 1 that more nodes are hired as AR increases for all EPNs, and when 5≤*H*<6, each EPN is by itself on a single node. Starting from *H*=6, triggers reduce the AR at some EPNs; consequently, nodes are gradually released except at *H*=7 when the rate reductions administered are not sufficient to release any node.

**Figure 4.**
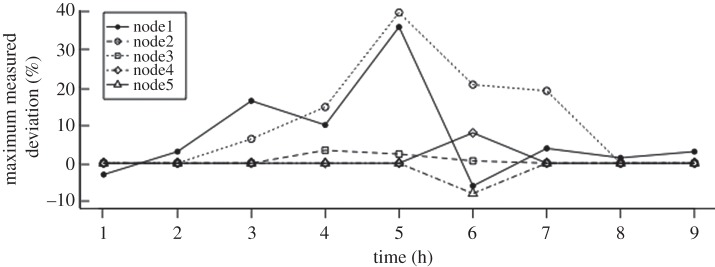
Deviation measurement in algorithm results.

Figure 4 plots, for each node, the maximum of *δ*^m^ of the EPNs hosted locally at *H*=1,2,…,8,9. (At *H*=9, AR_1_ was set to 0, bringing the system to an idle state.) We observe that HW (set to 20%) was exceeded during 4.3≤*H*≤6, the period when EPNs were receiving events at peak rates.

This observation leads to two inferences: an intelligent system such as CS is vital to ensure that RT meets *T* when EPNs are faced with the possibilities of event arrival rates fluctuating; secondly, nodes must report both RT and AR to CS in a proactive manner as proposed in §4, not just in response to significant changes they observe in AR. The latter arises owing to the fact that *W* tends to underestimate RT at large values of RT and hence adjustments to existing configuration are necessary even if a large AR remains constant for nearly an hour.

## 8. Conclusions

We have described a novel approach to deploying a collection of EPNs on cloud platforms in a manner such that the response times can be ensured to meet specified targets even when event arrival rates can fluctuate over time. Novelty of our approach lies in predicting response times based only on prior calibration and periodic measurements of event arrival rates and end-to-end latencies of EPNs; there is no need for intrusive, low-level measurements at operator level within EPNs.

Experiments confirm that prediction is reasonably accurate, and the heuristic for selecting appropriate configurations is effective. Periodic reporting of arrival rates and response times by EPNs is the only overhead imposed. This small but inevitable running overhead and the overhead of executing a heuristic selection algorithm make our approach a highly scalable one in managing a large number of EPNs with response time constraints on a cloud platform.

Recent work by Ishii & Suzumura [2] also addresses this practical problem of managing EPN response times by hiring enough virtual machines from a cloud infrastructure. Unlike us, they do not estimate the likely response times for the prevailing arrival rates but predict the likely arrival rates for deciding on the number of nodes needed, and this prediction is based on the prevailing rates and their variation trends. Their algorithm also takes the cost of hiring extra nodes as a parameter that we have not considered.

Currently, we are implementing the architecture to manage a distributed, large-scale system of 12 ambient kitchens in multiple locations each with 600 devices and hence requiring 600 EPNs. This would require addressing various limitations observed earlier, e.g. *W* underestimating RT and nodes being single core. We are currently developing formulae for multi-core machines as they are more common in practice. An important issue that has not been mentioned was the reliability of the (single) node hosting CS—whose functionality is central to our approach. This node must be made reliable against crashes and also secure against attacks, both of which can be accomplished using techniques presented in [10].
